# *(R)*-ketamine induces mGlu_5_ receptor-dependent antidepressant-like effects in the chronic unpredictable mild stress model of depression in mice

**DOI:** 10.1007/s00213-025-06803-0

**Published:** 2025-05-08

**Authors:** Agnieszka Pałucha-Poniewiera, Anna Rafało-Ulińska, Agata Faron-Górecka, Paulina Pabian, Katarzyna Kaczorowska

**Affiliations:** 1 Department of Neurobiology, Polish Academy of Sciences, Maj Institute of Pharmacology, 12 Smętna Street, 31-343 Kraków, Poland; 2 Department of Pharmacology, Maj Institute of Pharmacology, Polish Academy of Sciences, 12 Smętna Street, 31-343 Kraków, Poland; 3 Department of Medicinal Chemistry, Polish Academy of Sciences, Maj Institute of Pharmacology, 12 Smętna Street, 31-343 Kraków, Poland

**Keywords:** Antidepressant, BDNF, CUMS, EEF2, M-5MPEP, MGlu_5_ receptor, MTOR, RAAD, *(R)*-ketamine, TrkB

## Abstract

**Rationale:**

*(S)*-Ketamine, which is used to treat depression, has significant undesirable effects and has potential for abuse. A safe alternative to *(S)*-ketamine is *(R)*-ketamine. The relationship between *(R)*-ketamine and the mGlu_5_ receptor is unknown, although screening tests indicate the possibility of potentiation of the antidepressant effect of *(R)*-ketamine by the mGlu_5_ receptor negative allosteric modulator (NAM).

**Objectives:**

We aimed to investigate whether the antidepressant-like effect of *(R)*-ketamine is mGlu_5_ receptor-dependent. Specifically, we investigated the possibility of enhancing *(R)*-ketamine antidepressant-like effects using the partial mGlu_5_ receptor NAM, 2-(2-(3-methoxyphenyl)ethynyl)-5-methylpyridine (M-5MPEP), in a chronic unpredictable mild stress (CUMS) model of depression in mice.

**Methods:**

The effect of *(R)*-ketamine on mGlu_5_ receptor availability in the mouse brain was investigated using an autoradiographic method. Animal behaviors reflecting anhedonia, apathy, and helplessness were analyzed to study the rapid and sustained antidepressant-like effects of the combined administration of *(R)*-ketamine and M-5MPEP. Hippocampal protein levels were measured via Western blotting.

**Results:**

*(R)*-Ketamine altered mGlu_5_ receptor availability in several mouse brain regions. Importantly, in the hippocampus, *(R)*-ketamine reversed CUMS-induced effects. Behavioral studies revealed that M-5MPEP enhanced the effectiveness of a subeffective dose of *(R)*-ketamine. This drug combination effectively reduced CUMS-induced apathy- and anhedonia-like behavior symptoms. Changes in hippocampal eukaryotic elongation factor2 (eEF2) and tropomyosin receptor kinase B (TrkB) levels accompanied these effects.

**Conclusions:**

The weakening of mGlu_5_ receptor function in the hippocampus appears to be related to the *(R)*-ketamine antidepressant-like effect, and coadministration of the partial mGlu_5_ receptor NAM, M-5MPEP, might increase its antidepressant activity.

## Introduction

In recent years, ketamine has been widely studied for its particularly fast and long-lasting antidepressant effects. The substance referred to as ketamine is, in fact, a racemic mixture of *(R)*- and *(S)*-ketamine, and the latter enantiomer has been registered as a rapid-acting antidepressant drug (RAAD) for the treatment of treatment-resistant depression (TRD) (Mahase 2019). The two ketamine enantiomers differ in their mechanism of action at both the cellular and molecular levels (Fukumoto et al. 2017; Rafało-Ulińska and Pałucha-Poniewiera 2022; Yang et al. 2019; Shafique et al. 2024). This differentiation results in distinct strengths and durability of therapeutic effects and the occurrence and severity of adverse effects (Fukumoto et al. 2017; Chang et al. 2019; Rafało-Ulińska and Pałucha-Poniewiera 2022; Zhang et al. 2014). In general, *(S)*-ketamine causes dangerous, undesirable effects, including psychotomimetic, dissociative, and addictive effects, whereas the effects of *(R)*-ketamine are generally mild or even negligible (Chang et al. 2019; Rafało-Ulińska and Pałucha-Poniewiera 2022; Yang et al. 2015). Moreover, *(R)*-ketamine has been shown to induce antidepressant-like effects in animal tests and models of depression. Notably, preclinical data indicate that *(R)*-ketamine shows greater potency and longer-lasting antidepressant effects than *(S)*-ketamine does (Chang et al. 2019; Fukumoto et al. 2017; Rafało-Ulińska and Pałucha-Poniewiera 2022; Zhang et al. 2014). Clinical trials are only in the initial phases, although the results are promising for the treatment of both TRD and bipolar depression (Bandeira et al. 2023; Leal et al. 2021). According to Atai Life Sciences, *(R)*-ketamine, known in clinical trials as PCN-101, has demonstrated an encouraging safety profile and efficacy signals across all time points despite not achieving statistical significance at the primary endpoint. Research on the antidepressant effects of *(R)*-ketamine in humans is continuing (Shafique et al. 2024).

Studies aimed at explaining the mechanism of the antidepressant action of ketamine have shown that metabotropic glutamate (mGlu) receptors, especially mGlu_2/3_ and mGlu_5_ receptors, may play a potential role in this action (Elmeseiny and Müller 2024; Pałucha-Poniewiera 2022). Importantly, data on the possible role of mGlu_5_ receptors in the mechanism of action of ketamine have come from human studies, which have shown that intravenous ketamine affects mGlu_5_ receptor levels in depressed and healthy adults. For example, positron emission tomography (PET) has revealed a significant reduction in selective radioligand binding to the mGlu_5_ receptor in several brain regions after ketamine infusion (DeLorenzo et al. 2015; Esterlis et al. 2018). Despite the use of the same radioligand and imaging technique, ketamine- or *(S)*-ketamine-induced reductions in binding to the mGlu_5_ receptor have not been replicated in animals (Kosten et al. 2018; Müller Herde et al. 2018). Furthermore, no difference in mGlu_5_ transcript levels in the hippocampus or frontal cortex of rats was detected after *(S)*-ketamine administration (du Jardin et al. 2016). Only using prenatal stress as a depression model, which partially reflects a disease state, a significant role of the hippocampal mGlu_5_ receptor in mediating the rapid antidepressant-like effects of ketamine was demonstrated (Wang et al. 2020). Notably, no studies have addressed the effect of *(R)*-ketamine on mGlu_5_ receptor expression or availability.

A recent behavioral study supported the mGlu_5_ receptor-dependent mechanism of action of ketamine. It was found that the pharmacological suppression of the mGlu_5_ receptor was a condition for the effect of ketamine in the forced swim test (FST) in rats. The consequence of this relationship was that the mGlu_5_ receptor negative allosteric modulator (NAM), MTEP, enhanced the antidepressant-like effect of subanaesthetic ketamine (Gokalp and Unal 2024). Our recent studies, which used another screening test in mice, the tail suspension test (TST), revealed the possibility of enhancing the antidepressant effect of *(R)*-ketamine by the partial mGlu_5_ receptor NAM, 2-(2-(3-methoxyphenyl)ethynyl)−5-methylpyridine (M-5MPEP) (Pałucha-Poniewiera et al. 2024b), which has much greater potential as a drug compared to full NAMs, as it does not cause psychotomimetic effects or memory dysfunction (Gould et al. 2016; Holter et al. 2021). However, it should be noted that screening tests (such as the TST and FST) indicate therapeutic potential but do not reveal the dynamics of the effect and effectiveness in individuals with modeled depressive illness. These aspects can be examined using a depression model, e.g., chronic unpredictable mild stress (CUMS). Importantly, using the CUMS model in mice, we previously demonstrated a rapid and sustained antidepressant-like effect of *(R)*-ketamine (Rafało-Ulińska et al. 2022; Rafało-Ulińska and Pałucha-Poniewiera 2022), as well as the M-5MPEP effect (Pałucha-Poniewiera et al. 2024a).

Therefore, we investigated the effects of *(R)*-ketamine on mGlu_5_ receptor levels in various brain regions of mice in a CUMS model of depression and verified whether the observed effects on mGlu_5_ receptor levels are essential for the antidepressant-like efficacy of M-5MPEP. Following the changes in mGlu_5_ receptor availability and based on studies indicating the possibility of enhancing the antidepressant effect of *(R)*-ketamine by partial NAM of the mGlu_5_ receptor, we performed experiments using subthreshold doses of both substances individually and in combination to examine the occurrence of such enhancement in a depression model. For this purpose, we applied behavioral tests allowing for the examination of depression-like behaviors resembling the state of apathy, anhedonia and helplessness. In addition, we conducted experiments to determine which mechanisms may be responsible for such an effect. We explored two distinct theories explaining the mechanism of the rapid antidepressant action of ketamine (Kim et al. 2023): disinhibition theory, which suggests a crucial role of the mammalian target of rapamycin (mTOR) kinase (Li et al. 2010), and the theory of homeostatic synaptic plasticity, which posits crucial roles of eukaryotic elongation factor 2 (eEF2) kinase and brain-derived neurotrophic factor (BDNF)/tropomyosin receptor kinase B (TrkB) signaling (Autry et al. 2011). To this end, we investigated the influence of *(R)*-ketamine coadministered with M-5MPEP on the protein levels of mTOR, eEF2, BDNF and TrkB in the hippocampus of mice subjected to CUMS.

## Materials and methods

### Animals and housing

The experiments were performed on male C57BL/6 J mice (Charles River, Germany), 7 weeks of age at the beginning of the experiment. Animals were maintained under standard laboratory conditions (temperature 22 ± 2 °C, humidity 55 ± 10%, light phase 7:00–19:00) with free access to food and tap water. Two cohorts of mice designed for the CUMS model were established and housed in separate rooms: the non-stressed (NS) group and the CUMS group. Behavioral experiments were performed during the light period (8:00 am to 2:00 pm) by an observer unaware of the individual's affiliation to the experimental group. All procedures followed the European directive 2010/63/EU on the protection of animals used for scientific purposes and were approved by the Second Local Ethics Committee in Kraków, Poland (permission numbers: 69/2020 and 154/2023). The three Rs principles (Replacement, Reduction, and Refinement) were applied in the planning and execution of the experiments. Every effort was made to reduce the number of animals used and to avoid and minimize animal suffering.

### Compounds and treatment

M-5MPEP (2-[2-(3-methoxyphenyl)ethynyl]−5-methylpyridine, synthesized in the Department of Medicinal Chemistry, Maj Institute of Pharmacology Polish Academy of Sciences, by K.K.) (data on the synthesis, structure confirmation and the purity of the compound can be found in the supplementary materials of our previous study: Pałucha-Poniewiera et al. 2024a) and *(R)*-ketamine hydrochloride (Cayman Chemicals, Ann Arbor, USA) were diluted in a suspension of 0.5% methylcellulose/0.9% NaCl, which was used as a vehicle. Drug solutions were injected intraperitoneally (ip) at a constant 10 ml/kg volume. Doses and times of compounds administration were determined based on our own previous research and literature data (Pałucha-Poniewiera et al. 2024a, 2024b; Rafało-Ulińska and Pałucha-Poniewiera 2022; Yang et al. 2015).

### Chronic unpredictable mild stress

The CUMS procedure was performed based on our experience and previously published experimental schedules with the necessary modifications (Pałucha-Poniewiera et al. 2020, 2021). Briefly, after ten days of adaptation, the animals were subjected to the CUMS procedure for 3 weeks. Two stressors from those given below were applied daily:

- Restraint stress (60 min)
- Cage tilting (45°) (6 h)
- Wet bedding (2 h)
- Removal of sawdust (2 h)
- Placing a mouse in the empty cage of another mouse (2 h)
- Reversed light–dark cycle (48 h)
- Substitution of sawdust with 37° C water (60 min)
- 3–6 individuals in a cage with 37° C water (60 min)
- Overcrowding (20–24 individuals) (60 min)

The NS group was not subjected to any procedure during this time except for weighing and handling animals. On the 21 st day from the beginning of CUMS, the tested compounds were administered ip. Further procedures were performed according to the requirements of the specific experimental schedules. All details covering the times from compound administration to the execution of the tests are given in each figure presenting the results.

### Autoradiographic analysis of [^3^H]MPEP binding

Coronal brain Sects. (12 μm) were prepared using a Leica Jung CM 3000 cryostat microtome (Leica, Germany) at the following bregma levels: 2.80 mm, 1.34 mm, −1.94 mm, −3.08 mm, and −4.36 mm, based on the Mouse Brain Atlas (Paxinos and Franklin, 2001). The slices were mounted on a gelatine-covered microscope slide and stored at −20 °C.

The slides were preincubated for 15 min at room temperature in Buffer B1 (50 mM HEPES with 3 mM MgCl₂, pH 7.4) to remove endogenous ligands. This was followed by incubation for 1 h at room temperature in the same buffer containing 10 nM [^3^H]MPEP (2-Methyl-6-(phenylethynyl)pyridine; Hartmann Analytic, specific activity: 60 Ci/mmol). To determine nonspecific binding, an analogous set of slides was incubated with slides was incubated with 10 µM MTEP (3-((2-Methyl-1,3-thiazol-4-yl)ethynyl)pyridine; Tocris) in addition to [^3^H]MPEP, as described by Varnas et al., 2018, with modifications. After incubation, slides were washed twice in cold Buffer B1 for 5 min each, followed by a single rinse in cold distilled water. Slides were then air-dried for 24 h at room temperature. Dried slides were exposed to an imaging plate (Fujifilm, Japan) with autoradiography microscales (GE Healthcare) for 7 days. The resulting autoradiograms were analyzed and quantified using ImageGauge software (Fujifilm, Japan).

## Behavioral tests

### Tail suspension test

The experiments were carried out as previously described (Pałucha-Poniewiera et al. 2020). The mice were habituated to the testing room for 30 min before the experiments. Each mouse was attached by its tail to the table's edge with adhesive tape. The total duration of immobility was assessed for six minutes.

### Splash test

The splash test was performed as described previously (Pałucha-Poniewiera et al. 2021) with minor modifications. Briefly, animals were adapted for the experimental room for 30 min. The test was performed under dimmed lighting. A high-viscosity 10% sucrose solution (approximately 0.2 ml) was sprayed on the dorsal coat of the mice to stimulate self-grooming behavior. Then, the duration of grooming was recorded for five minutes.

### Sucrose preference test (SPT)

The SPT was performed as previously described (Pałucha-Poniewiera et al. 2021). For 24 h, the animals could choose between one of the two identical bottles. The first bottle contained a 1% sucrose solution, and the second contained tap water. The position of the bottles was switched 12 h after the start of the experiment. At the beginning and end of the test, the bottles were weighed, and the liquid consumption was calculated. The preference for sucrose consumption was calculated as a ratio of the consumed sucrose solution to the total amount of liquid consumed.

### Locomotor activity

12-station photobeam activity system (Opto Varimex 4, Auto Track System 4.41, Columbus Instruments, Columbus, OH, USA) equipped with Plexiglas locomotor activity chambers (40 × 40 × 40 cm) was used to measure the locomotor activity of the mice. After placing the animals individually in the locomotor activity chambers, the total distance traveled during a 30-min experimental session was measured and stored every 6 min.

### Synaptosome preparation and western blotting

Tissue samples were dissected from the hippocampus and frozen on dry ice. After thawing on ice, the samples were homogenized in ice-cold lysis buffer [0.32 M sucrose, 20 mM HEPES (pH 7.4), 1 mM EDTA; 1 × protease inhibitor cocktail, 5 mM NaF, and 1 mM NaVO3]. Homogenates were centrifuged at 2800 rpm for 10 min at 4 °C, and the resulting supernatant was centrifuged at 12,000 rpm for 10 min at 4 °C. The obtained pellets were then sonicated in protein lysis buffer (50 mM Tris–HCl (pH 7.5), 150 mM NaCl, 1% Triton X-100, 0.1% SDS, 2 mM EDTA, 1 mM NaVO3, 5 mM NaF, and protease inhibitor cocktail). BCA kit (Thermo Scientific, USA) was used to measure protein concentrations. The proteins were separated by SDS-PAGE and transferred to nitrocellulose membranes. 1% of the blocking solution (BM Chemiluminescence Western Blotting Kit (Mouse/Rabbit) made by Roche, Switzerland) was used to block for 1 h. Then, the membranes were incubated overnight at 4 °C with the primary antibodies: Anti-mTOR (mTOR 1:1000; Cell Signaling Technology, USA), Anti-phospho-mTOR (pmTOR, S2481, 1:1000; Abcam, USA), Anti-eEF2 (eEF2 1:1000; Abcam, USA), Anti- phospho-eEF2 (pheEF2 (phospho T56) 1:1000; Abcam, USA), Anti-Phospho-TrkB (pheTrkB 1:1000; Sigma-Aldrich, Germany), Anti-TrkB (TrkB 1:1000; Cell signaling Technology, USA), anti-BDNF (BDNF 1:1000, Abcam, USA). Afterward, the membranes were washed three times for 10 min using Tris-buffered saline with Tween (TBS-T) and incubated for 60 min with secondary antibodies (anti-mouse or anti-rabbit-IgG-peroxidase-conjugated antibodies Vector Laboratories, USA). After incubation, the membranes were washed thrice for 10 min with TBS-T and incubated with a detection reagent (Bio-Rad, USA). Fuji-Las 1000 system, equipped with Fuji Image Gauge v.4.0 software, was used to visualize and measure the signal. A primary monoclonal antibody, Glyceraldehyde 3-phosphate dehydrogenase (GAPDH, 1:500; Millipore, Germany), was used to check the transfer and loading. The final result is the ratio of particular proteins’ optical density to GAPDH’s optical density.

## Results

### Effect of (R)-ketamine treatment on the [^3^H]MPEP binding to the mGlu_5_ receptor in the CUMS model of depression

**Fig. 1:**
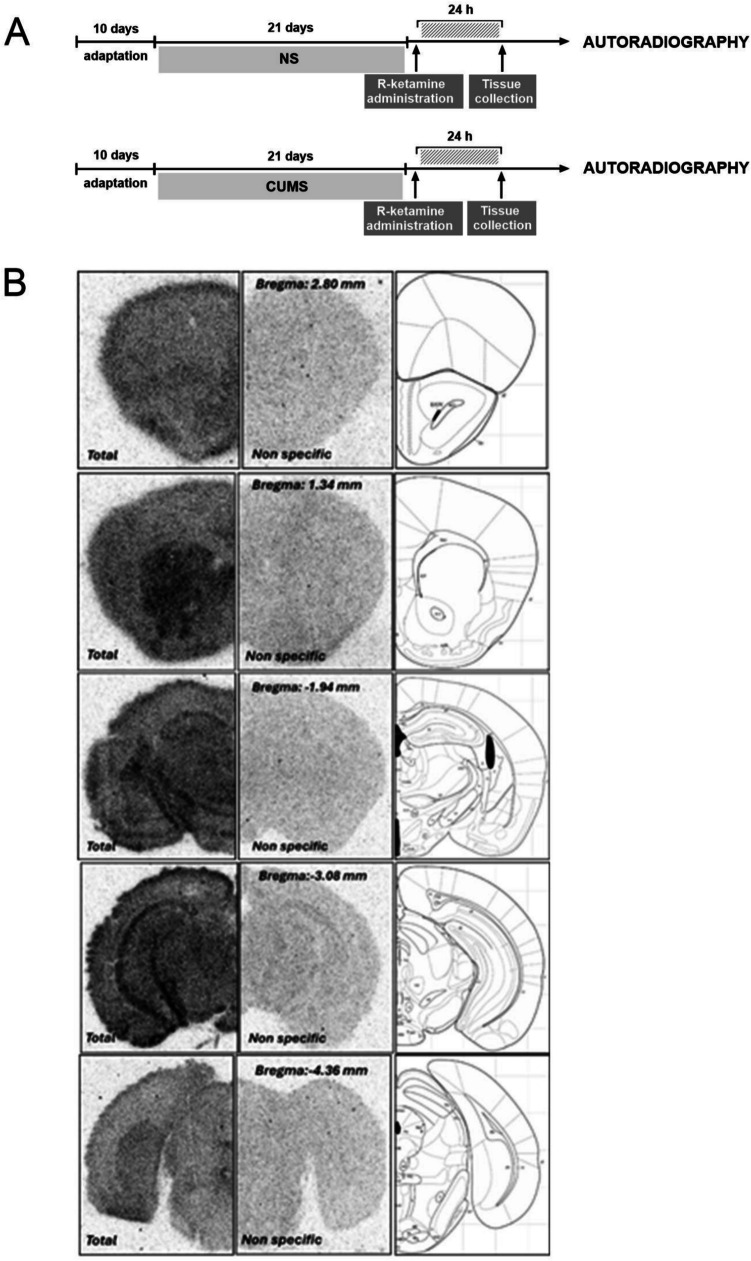

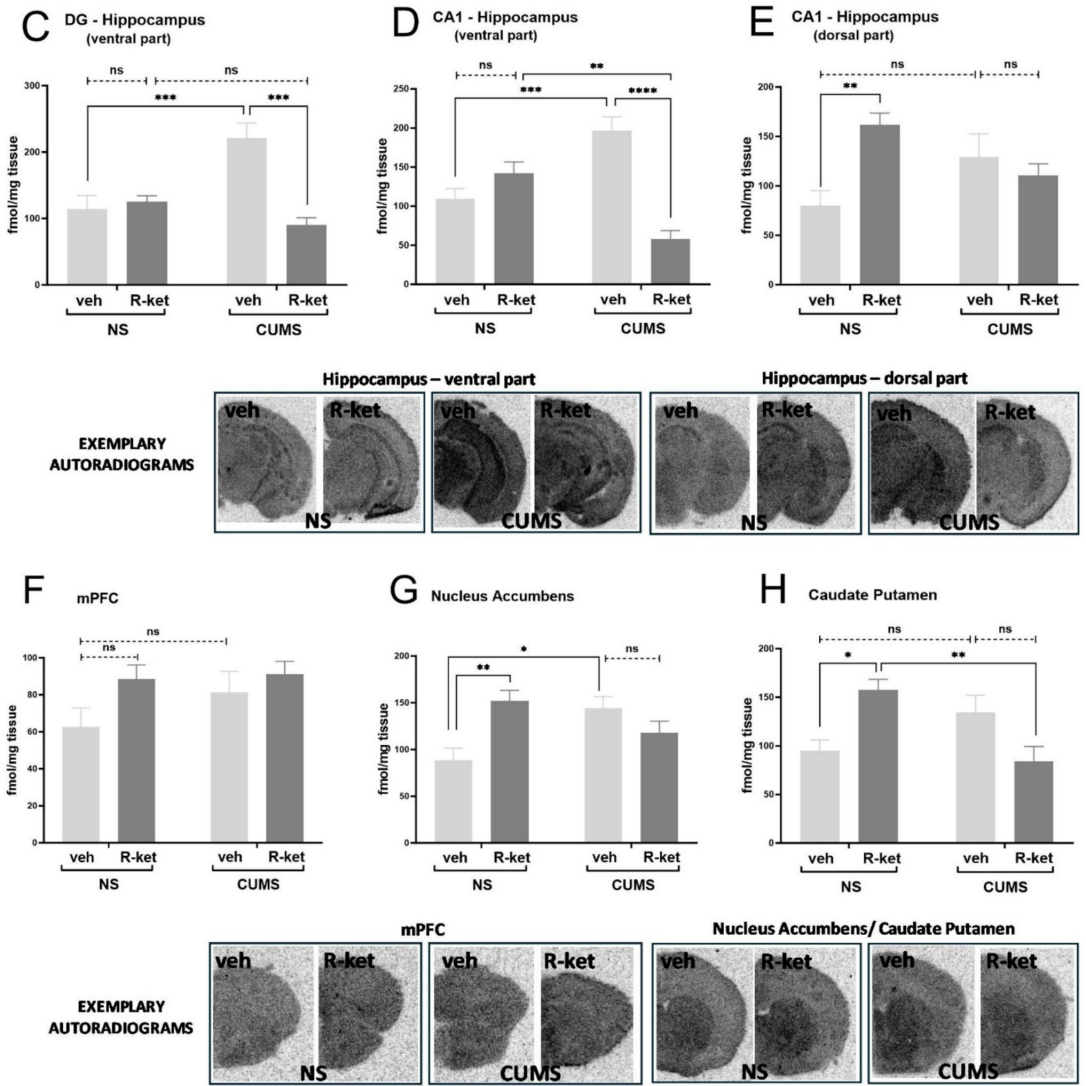
The effect of *(R)*-ketamine (10 mg/kg) treatment on the [^3^H]MPEP binding to the mGlu_5_ receptor in the CUMS model of depression. **(A)** scheme of the experiment; **(B)** representative autoradiograms showing [^3^H]MPEP binding in mouse brain sections. The left panel displays total binding; the middle panel shows nonspecific binding (blocked by 10 µM MTEP); the right panel presents the corresponding section from the Paxinos & Franklin atlas (Paxinos and Franklin 2021); **(C)** effect in a ventral part of the dentate gyrus (DG) region of the hippocampus; **(D)** effect in a ventral part of the CA1 region of the hippocampus; **(E)** effect in a dorsal part of the CA1 region of the hippocampus; **(F)** effect in the mPFC; **(G)** effect in the nucleus accumbens; **(H)** effect in the caudate putamen. The values are expressed as the means ± SEM and were analyzed by two-way ANOVA followed by Tukey’s post hoc test. * *p* < 0.05, ** *p* < 0.01, *** *p* < 0.001, **** *p* < 0.0001 vs. a respective vehicle group; ns—not statistically significant

An autoradiographic analysis of mGlu_5_ receptors using [^3^H]MPEP radioligand showed its specific binding in several brain regions, including the hippocampus, mPFC, nucleus accumbens, and caudate putamen (Fig. 1B). Two-way ANOVA revealed a significant increase in [^3^H]MPEP binding in CUMS mice compared to NS controls in a ventral part of the *dentate gyrus* (DG) region of the hippocampus (*p* < 0.001; Tukey's multiple comparisons test). Furthermore, it was found that *(R)*-ketamine decreased the CUMS-induced effect in this region (CUMS x *(R)*-ketamine interaction: [F(1,24) = 16.28, *p* < 0.001]) and Tukey's post-hoc test revealed a significant difference between *(R)*-ketamine-treated and vehicle-treated CUMS mice (p < 0.001) (Fig. 1C). The similar results were obtained when analyzing ventral part of the CA1 region of hippocampus. An increase in [^3^H]MPEP binding was observed in CUMS animals in this region (*p* < 0.001; Tukey's test), and *(R)*-ketamine decreased this effect (*p* < 0.0001; Tukey's test). Two-way ANOVA also revealed an interaction between CUMS and (*R)*-ketamine [F(1,32) = 32.43, *p* < 0.001] (Fig. 1D). On the other hand, in the dorsal part of the hippocampal CA1 region, the effect of CUMS on [^3^H]MPEP binding was insignificant, and the effect of *(R)*-ketamine was observed only in NS mice (*p* < 0.01; Tukey's test) (Fig. 1E). An autoradiographic analysis of mGlu_5_ receptors in the mPFC showed no significant effects of CUMS or *(R)*-ketamine in any experimental group (NS or CUMS) (*p* > 0.05; Tukey's test) (Fig. 1F). In contrast, in the *nucleus accumbens*, an increased [^3^H]MPEP binding was observed in CUMS mice, compared to NS controls (*p* < 0.05; Tukey's test), and no significant difference was found between *(R)*-ketamine-treated and vehicle-treated CUMS animals (*p* > 0.05; Tukey's test). However, a trend to reduce *[*^*3*^*H]MPEP* binding was seen in the CUMS group, while in the NS group, *(R)*-ketamine significantly increased this parameter (*p* < 0.01; Tukey's test), thus inducing an opposite effect in NS and CUMS mice (CUMS x *(R)*-ketamine interaction: [F(1,28) = 12.52, *p* < 0.01]) (Fig. 1G). In *caudate putamen*, CUMS did not induce changes in *[*^*3*^*H]MPEP* binding (*p* > 0.05, Tukey's test). At the same time, *(R)*-ketamine treatment increased this parameter in NS mice (*p* < 0.05; Tukey's test) while not inducing similar effects in the CUMS group (*p* > 0.05, Tukey's test) (Fig. 1H).

### Acute effects of M-5MPEP in the TST and locomotor activity test in the CUMS model of depression

**Fig. 2:**
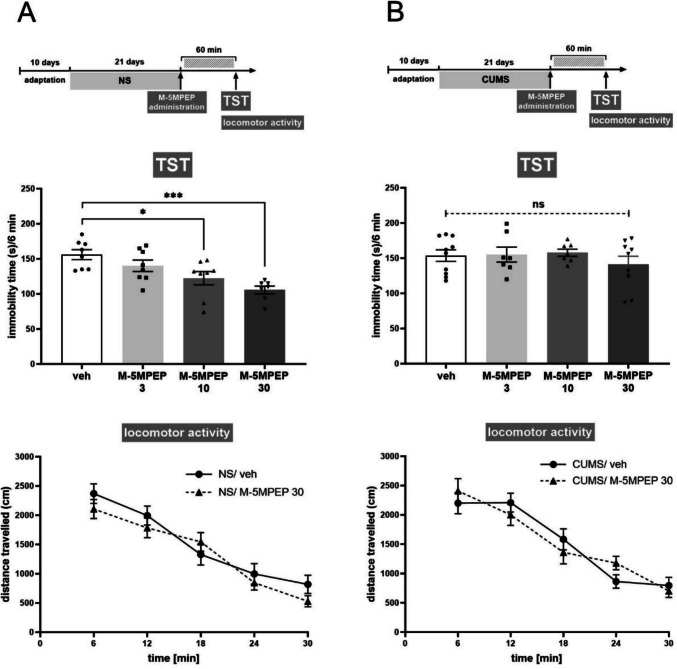
Acute effects of M-5MPEP (3–30 mg/kg) in the tail suspension test and locomotor activity test in the CUMS model of depression. (A) effects M-5MPEP, 60 min after ip administration in the NS group; (B) effects of M-5MPEP 60 min after ip administration in the CUMS group. The values are expressed as the means ± SEM and were analyzed by one-way ANOVA followed by Dunnett's post hoc test (TST) or repeated-measures ANOVA (locomotor activity). * *p* < 0.05, *** *p* < 0.001 vs. vehicle group; ns—not statistically significant

In NS mice, acute administration of the mGlu_5_ receptor NAM, M-5 MPEP (3–30 mg/kg), produced a dose-dependent antidepressant-like effect in the TST 60 min after ip injection [F(3,27) = 7.504, *p* < 0.001). Dunnett's multiple comparisons test showed that this effect was significant for 10 and 30 mg/kg (*p* < 0.05 and *p* < 0.001, respectively) (Fig. 2A). At the same time, the highest dose used (30 mg/kg) did not affect the locomotor activity of NS animals [F(1,70) = 1.975, *p* > 0.05], and Bonferroni's multiple comparisons test showed no difference between the groups at any tested time-point (*p* > 0.05) (Fig. 2A). On the other hand, in the CUMS group, M-5MPEP, administered in the same dose range, did not induce any effects in the TST 60 min after administration [F(3,29) = 0.6225, *p* > 0.05] (Fig. 2B). Locomotor activity test also showed no effect of M-5MPEP (30 mg/kg) on the behavior of mice F(1,70) = 1.330e-005, *p* > 0.05] (Fig. 2B).

### Sustained antidepressant-like effects of a combined administration of subeffective doses of (R)-ketamine and M-5MPEP in the CUMS model of depression

**Fig. 3:**
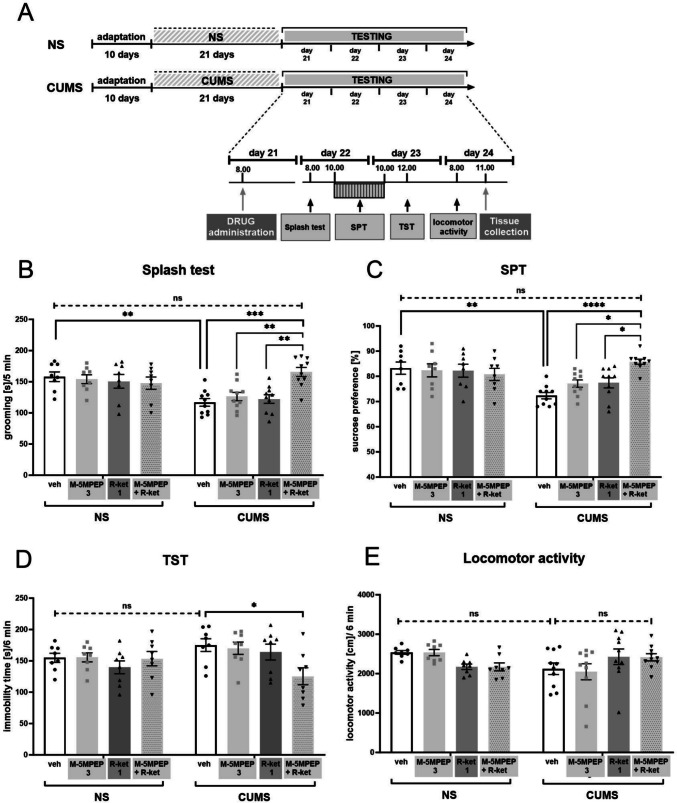
Sustained antidepressant-like effects of a combined administration of subeffective doses of *(R)*-ketamine (1 mg/kg) and M-5MPEP (3 mg/kg) in the CUMS model of depression. **(A)** scheme of the experiment; **(B)** effect in the splash test; **(C)** effect in the sucrose preference test; **(D)** effect in the tail suspension test; **(E)** effect in the locomotor activity test. The values are expressed as the means ± SEM and were analyzed by three-way ANOVA followed by Tukey’s post hoc test. * *p* < 0.05, ** *p* < 0.01, *** *p* < 0.001, **** *p* < 0.0001 vs. a respective control; ns—not statistically significant

In the splash test, a significant reduction in grooming time was demonstrated in CUMS mice compared to NS mice, indicating a stress-induced apathy-like state (CUMS effect [F(1,32) = 11.66, *p* < 0.01]) (Fig. 3B). Tukey's multiple comparisons tests confirmed the deference between NS/veh vs. CUMS/veh group (*p* < 0.01) and showed that combined administration of *(R)*-ketamine (1 mg/kg) and M-5MPEP (3 mg/kg) had no effect in NS mice (*p* > 0.05) but reversed the CUMS-induced reduction in grooming (*p* < 0.001). In contrast, the compounds administered alone did not alter grooming time in CUMS mice (*p* > 0.05) (Fig. 3B).

The SPT test also showed a significant effect of CUMS on the tested parameter, indicating anhedonia induced by chronic stress (CUMS effect F(1,32) = 7.019, *p* < 0.05]) (Fig. 3C). Additionally, Tukey's multiple comparisons test showed a significant reduction in sucrose preference in vehicle-treated CUMS mice compared to NS controls (*p* < 0.01). It was further demonstrated that in CUMS animals, an inactive dose of *(R)*-ketamine (1 mg/kg) given concomitantly with an inactive dose of M-5MPEP (3 mg/kg) significantly reduced stress-induced anhedonia (*p* < 0.0001) (Fig. 3C).

In turn, the TST did not show a significant effect of CUMS on the parameter studied (CUMS effect [F(1,28) = 1.356, *p* > 0.05]), although a tendency to prolong immobility time could be observed in stressed mice (Fig. 3D). However, in CUMS animals, it was shown that the combined administration of subthreshold doses of *(R)*-ketamine and M-5MPEP significantly reduced the immobility time of the animals (*p* < 0.05), indicating an antidepressant-like effect. There was no such effect in non-stressed animals (*p* > 0.05) (Fig. 3D).

Statistical analysis of the results obtained in the locomotor activity test showed no influence of CUMS [F (1,32) = 1.131, *p* > 0.05] or any tested factor on the locomotor activity of mice (*p* > 0.05) (Fig. 3E).

### Effect of a combined administration of subeffective doses of (R)-ketamine and M-5MPEP on the level of mTOR, eEF2, TrkB, and BDNF in the hippocampus in the CUMS model of depression

**Fig. 4:**
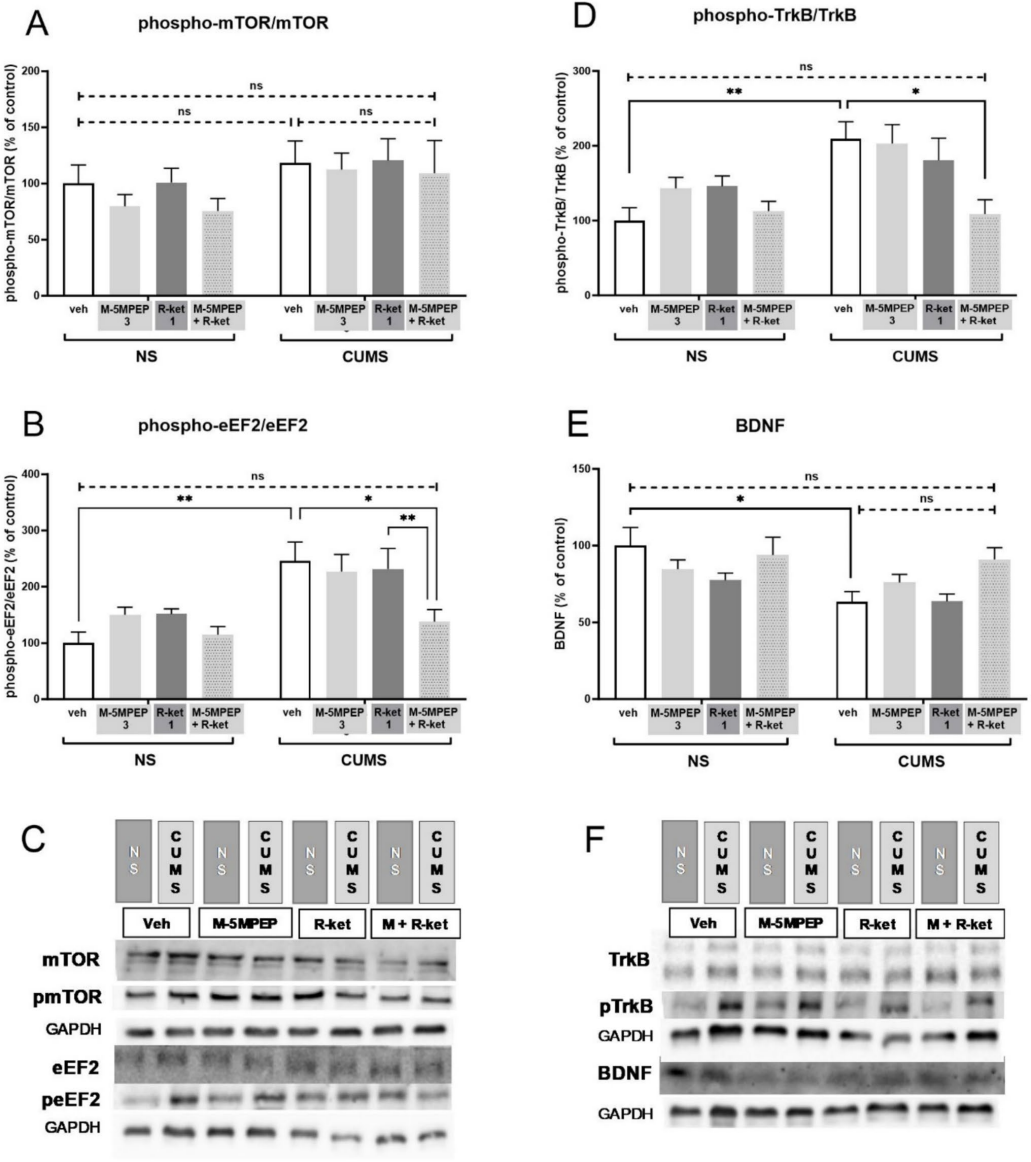
The effects of a combined administration of subeffective doses of *(R)*-ketamine (1 mg/kg) and M-5MPEP (3 mg/kg) on the mTOR **(A)**, eEF2 **(B)**, TrkB **(D)**, and BDNF **(E)** levels determined by Western blot analysis in the hippocampus in the CUMS model of depression. **(C)** the exemplary blot for charts A and B; **(F)** the exemplary blots for charts D and E. The data were analyzed using three-way ANOVA followed by Tukey’s post hoc test. Values (the mean ± SEM) are expressed as percentage of changes vs. control levels. * *p* < 0.05, ** *p* < 0.01 vs. a respective control; ns—not statistically significant

Three-way ANOVA followed by Tukey's test, used to analyze three factors influencing the results (CUMS x M-5MPEP x *(R)*-ketamine), did not reveal any differences in the phospho-mTOR/mTOR ratio between the tested experimental groups (*p* > 0.05) (Fig. 4A). In contrast, hippocampal phospho-eEF2/eEF2 ratio was higher in CUMS mice compared to non-stressed animals [F(1,36) = 13,69, *p* < 0.001] and Tukey's post-hoc test showed the difference between NS/veh vs. CUMS/veh group (*p* < 0.01). Furthermore, combined administration of *(R)*-ketamine (1 mg/kg) and M-5MPEP (3 mg/kg) reversed the CUMS-induced increase in phospho-eEF2/eEF2 ratio (*p* < 0.05). This result was not statistically different from the non-stressed control group (*p* > 0.05) (Fig. 4B). Statistical difference was also found between phospho-TrkB/TrkB ratio in CUMS mice vs. NS mice [F(1,36) = 9.572, *p* < 0.01]. Tukey's multiple comparisons test revealed this effect (*p* < 0.01). Moreover, combined administration of *(R)*-ketamine (1 mg/kg) and M-5MPEP (3 mg/kg) lowered the CUMS-induced effect (*p* < 0.05, Tukey's test) to the level not statistically different from the level of control non-stressed animals (*p* > 0.05, Tukey's test) (Fig. 4D). In the case of BDNF, the effect to decrease its hippocampal level was found in CUMS mice [F(1,36) = 7,198, *p* < 0.05]. The treatment with a combination of tested drugs did not statistically influence this effect (*p* > 0.05, Tukey's test). However, a distinct trend to increase BDNF levels in drugs-treated CUMS animals vs. vehicle-treated animals was observed. Of note, BDNF level in CUMS mice treated with (*R)*-ketamine (1 mg/kg) and M-5MPEP (3 mg/kg) was not different from the level in non-stressed controls (*p* > 0.05, Tukey's test) (Fig. 4E).

## Discussion

The results of the present study indicate that *(R)*-ketamine administered at antidepressant-relevant doses (Rafało-Ulińska et al. 2022; Yang et al. 2019) altered mGlu_5_ receptor availability in selected brain structures, generally causing an increase in nonstressed mice and a decrease in mice subjected to CUMS. Importantly, in the hippocampus (specifically in the dentate gyrus and ventral part of the CA1 region), an antidepressant dose of *(R)*-ketamine reversed the stress-induced increase in binding of the mGlu_5_ receptor ligand, suggesting that the hippocampus may play a specific role in the mechanism of antidepressant action of *(R)*-ketamine related to mGlu_5_ receptor downregulation. The hippocampus is a brain region involved in emotion, learning, and motivation and is strongly implicated in the pathophysiology of depression. It is also a key brain structure involved in the mechanism of the antidepressant action of ketamine, as suggested by the previously mentioned theory of homeostatic synaptic plasticity (Kim et al. 2023). The hippocampus is also an area with a high mGlu_5_ receptor density (Shigemoto et al. 1993); the mGlu_5_ receptor is involved in the regulation of glutamatergic transmission and engages in close functional cooperation with the NMDA receptor (mGlu_5_ receptor agonists enhance NMDA receptor functions, and mGlu_5_ receptor antagonists weaken NMDA receptor functions) (Tu et al. 1999). Based on the above data and our results, we suggest that in the chronic stress-based animal model of depression (CUMS), in which mGlu_5_ receptor availability in the hippocampus is increased, the action of *(R)*-ketamine may be related, at least in part, to the reduction in the functional availability of the mGlu_5_ receptor via a mechanism that remains to be further elucidated. Interestingly, no significant changes in mGlu_5_ receptor availability were detected in the mPFC, the brain region involved in the mechanism of the antidepressant effects of both ketamine and its enantiomers (Li et al. 2010; Yang et al. 2015; Zhang et al. 2019). Thus, the antidepressant effect of *(R)*-ketamine in the mPFC does not appear to be related to the regulation of mGlu_5_ receptor functions.

Our results are partially in line with those of Wang et al. (2020), who demonstrated that prenatal stress-induced pro-depressive-like behavior in rats was correlated with an increase in hippocampal mGlu_5_ levels in offspring susceptible to stress. Furthermore, ketamine significantly reversed stress-induced changes in rat behavior and mGlu_5_ expression. Notably, the same study revealed that the overexpression of the hippocampal mGlu_5_ receptor in wild-type rats resulted in depression-like behavior that was abolished by ketamine. In contrast, knockdown of hippocampal mGlu_5_ decreased depression-like behavior in rats susceptible to prenatal stress (Wang et al. 2000). Thus, the *(R)*-ketamine used in the mouse CUMS model appears to induce an action similar to the ketamine effect in the rat prenatal stress model, which strengthens the hypothesis that the mGlu_5_ receptor plays a significant role in the mechanism of the antidepressant action of ketamine and its enantiomer, *(R)*-ketamine.

Our research is also in line with human studies using the PET technique, which revealed a significant ketamine-induced reduction in mGlu_5_ receptor availability in most brain regions assessed in depressive patients (Esterlis et al. 2018). These changes persisted 24 h after ketamine administration. Notably, a correlation between the antidepressant response to ketamine and reduced radioligand binding to the mGlu_5_ receptor was found only in the hippocampus, not in the PFC, strongly suggesting the involvement of the hippocampal mGlu_5_ receptor in the mechanism of the antidepressant action of ketamine (Esterlis et al. 2018) and thus confirming our observations.

Because an increase in mGlu_5_ receptor availability in CUMS mice was observed in most of the analyzed brain structures, we investigated whether this effect impacts the antidepressant-like efficacy of the mGlu_5_ receptor ligand in the screening test. A partial NAM of the mGlu_5_ receptor, M-5MPEP, was chosen for this study. We found that M-5MPEP induced a dose-dependent antidepressant-like effect during the TST 60 min after administration in the nonstressed group of mice without affecting locomotor activity, confirming our previously published data (Pałucha-Poniewiera et al. 2024b). However, in mice subjected to CUMS, M-5MPEP was inactive during the TST, although the experimental conditions were carefully replicated. Since acute drug effects during the TST reflect receptor-dependent neurotransmission actions rather than neuroplastic changes, we speculate that the lack of effects of M-5MPEP during the TST in CUMS mice might be due to an insufficient dose to block the receptor, the expression of which is much greater in stressed mice. The ethical restrictions imposed by the ethics committee prevented us from using higher doses of the compound in this experiment, as this would have required a chronic stress procedure in an additional group of animals. Regardless, our results may serve as a caution against comparing the efficacy of drugs in animals subjected to stressful procedures versus nonstressed controls, as the dose‒response curves may be shifted due to changes in the availability of receptors responsible for the behavioral response.

Since the decrease in mGlu_5_ receptor availability after *(R)*-ketamine administration in the CUMS model of depression appears to be related to its antidepressant-like action, we investigated whether the effect of *(R)*-ketamine would be enhanced by coadministration of an mGlu_5_ receptor antagonist, which could additionally reduce the activity of this receptor. This hypothesis was based on the well-documented antidepressant-like effects of various mGlu_5_ receptor NAMs, which have shown activity not only in screening tests but also in animal models of depression (Chaki et al. 2013; Fuxe and Borroto-Escuela 2015; Kato et al. 2015; Pałucha et al. 2005). For this study, we again selected a partial mGlu_5_ receptor NAM, M-5MPEP, a compound with high therapeutic potential that shows activity in the CUMS model 24 h after short-term, 4-day administration, to a degree comparable to that of *(S)*-ketamine (Pałucha-Poniewiera et al. 2024a), and we used an analogous regimen in this study.

We showed the enhancement of a subthreshold dose of *(R)*-ketamine by coadministration of a subthreshold dose of M-5MPEP on all the studied behavioral parameters used to assess the antidepressant-like effect 24 h after the last administration, which is characteristic of RAAD. Compared with untreated anhedonic mice subjected to stress, CUMS model mice treated with *(R)*-ketamine coadministered with M-5MPEP presented a decrease in the stress-induced apathy-like state in the splash test and an increase in the preference for sucrose consumption. Additionally, we observed a decrease in the stress-enhanced immobility time during the TST, which was performed 2 days after drug administration. Importantly, all the observed effects occurred only in the groups subjected to the CUMS procedure, and there was no effect in the NS controls, which may suggest the specificity of the effect. The possible effect of the drug combination on sedation or hyperlocomotion, which could have influenced the false-positive assessment of behavioral effects in the tests used, was also excluded, as no changes in spontaneous locomotor activity were observed between the groups of animals studied.

The potentiation of the antidepressant effect of *(R)*-ketamine by the partial mGlu_5_ receptor NAM may have practical implications, including lowering the therapeutic dose of this compound and, simultaneously, reducing the probability of adverse effects.

Notably, our results confirm previous data on enhancing ketamine or *(R)*-ketamine action by mGlu_5_ receptor NAMs (MTEP, M-5MPEP) in screening tests (the TST, FST) and extend these findings with new data from a depression model (Gokalp and Unal 2024; Pałucha-Poniewiera et al. 2024b).

Analyses of hippocampi revealed that neither CUMS nor the tested compounds influenced mTOR phosphorylation, indicating a lack of a role of this kinase in the mechanism of the antidepressant-like action of the tested substances. However, according to the disinhibition theory, which indicates that mTOR is the main factor regulating neuroplastic processes in the mechanism of action of ketamine, these processes have been described in the mPFC (Li et al. 2010). We selected the hippocampus for Western blot analysis since our autoradiographic results and human data showed that this area regulates the mGlu_5_ receptor level by *(R)*-ketamine. In addition, the different mechanisms of action of *(S)*-ketamine and *(R)*-ketamine should be considered. The results obtained in this study are consistent with our previous observations using the CUMS model, indicating that *(R)*-ketamine does not affect the activation of mTOR in the hippocampus (Rafało-Ulińska et al. 2022). Additionally, in another animal model of depression, namely, social defeat stress (SDS), mTOR does not play a role in the antidepressant effect of *(R)*-ketamine, in contrast to *(S)*-ketamine, the action of which is dependent on the activation of this kinase (Yang et al. 2018).

In contrast, we observed a significant increase in the phospho-eEF2/eEF2 ratio under the influence of chronic stress and a statistically significant decrease in this effect in animals that were coadministered *(R)*-ketamine and M-5MPEP, which may indicate the involvement of eEF2 kinase in the mechanism of their antidepressant action. Phosphorylation of eEF2 suppresses protein translation (Sutton et al. 2007). In the hippocampus, eEF2 was shown to play a crucial role in the regulation of ketamine-induced neuroplasticity involving the upregulation of several key proteins, such as BDNF, which is required for the rapid antidepressant effect of ketamine (Autry et al. 2011). Therefore, the increased eEF2 phosphorylation in CUMS-induced animals in our study may be related to impairments in neuroplasticity processes. However, the ability of *(R)*-ketamine and M-5MPEP coadministration to reduce the phospho-eEF2/eEF2 ratio in CUMS mice may be associated with their antidepressant-like activity, which is related to increased protein translation. To our knowledge, this is the first report on the role of eEF2 phosphorylation in the mechanism of action of *(R)*-ketamine, specifically, coadministration with M-5MPEP.

One of the key products of eEF2-regulated translation in the hippocampus is BDNF (Kim et al. 2023), and BDNF/TrkB signaling may be essential for the antidepressant effect of *(R)*-ketamine. For example, *(R)*-ketamine induced a beneficial effect on BDNF/TrkB signaling in the SDS model of depression, which correlated with an antidepressant-like effect and was more potent than *(S)*-ketamine (Yang et al. 2015). Moreover, in the CUMS model, sustained *(R)*-ketamine anti-anhedonic and anti-apathetic effects were blocked by prior administration of a TrkB receptor antagonist, ANA-12 (Rafało-Ulińska and Pałucha-Poniewiera 2022). Here, we observed a decrease in BDNF in the hippocampus under CUMS conditions, which supports previous findings in a CUMS model in rats (Sun et al. 2016). Moreover, the combined administration of *(R)*-ketamine and M-5MPEP did not significantly reverse this effect, although a trend toward an increase in BDNF levels was observed. However, we must consider that the whole hippocampus was used for Western blot studies, so the analysis did not consider local changes in BDNF levels in different regions. This may be one reason for the lack of an apparent statistically significant change in the level of this protein. In addition, a significant increase in the phosphorylation of the TrkB protein, the receptor for BDNF, was observed in untreated animals subjected to stress. Importantly, this effect was significantly reversed by the combined administration of *(R)*-ketamine and M-5MPEP. The CUMS-induced increase in TrkB phosphorylation may be an adaptive change in response to reduced levels of BDNF caused by stress. Therefore, the reduction in the phospho-TrkB/TrkB ratio under the influence of the tested drugs in CUMS animals may indicate normalization of BDNF levels and restoration of BDNF/TrkB signaling to a normal state. However, when another model of depression, namely, the SDS model in mice, was used, a stress-induced decrease in both BDNF and TrkB was observed, indicating a general decrease in BDNF/TrkB signaling activity (Yang et al. 2015). We surmise that different animal models and experimental conditions may be the cause of the opposite results.

Overall, we conclude that an antidepressant dose of *(R)*-ketamine changes mGlu_5_ receptor availability in the mouse brain in a CUMS model of depression. Notably, *(R)*-ketamine reversed the stress-induced increase in mGlu_5_ receptor availability in the hippocampus, which may indicate that the reduction in mGlu_5_ receptor activity in this structure plays an important role in the mechanism of the antidepressant action of *(R)*-ketamine. This assumption was further confirmed by the behavioral results, indicating the intensification of a subthreshold dose of *(R)*-ketamine by coadministration of the partial NAM of the mGlu_5_ receptor, M-5MPEP. This effect not only indicates the involvement of the mGlu_5_ receptor in the mechanism of action of *(R)*-ketamine but also may have a practical application, as it paves the way to improve the treatment of depression.

## Data Availability

All relevant data are presented in the manuscript; raw data are available upon request from the corresponding author.
